# Liver lipophagy ameliorates nonalcoholic steatohepatitis through extracellular lipid secretion

**DOI:** 10.1038/s41467-023-39404-6

**Published:** 2023-07-13

**Authors:** Yoshito Minami, Atsushi Hoshino, Yusuke Higuchi, Masahide Hamaguchi, Yusaku Kaneko, Yuhei Kirita, Shunta Taminishi, Toshiyuki Nishiji, Akiyuki Taruno, Michiaki Fukui, Zoltan Arany, Satoaki Matoba

**Affiliations:** 1 Department of Cardiovascular Medicine, Graduate School of Medical Science, Kyoto Prefectural University of Medicine, Kyoto, 602-8566 Japan; 2 Department of Endocrinology and Metabolism, Graduate School of Medical Science, Kyoto Prefectural University of Medicine, Kyoto, 602-8566 Japan; 3 Department of Nephrology, Graduate School of Medical Science, Kyoto Prefectural University of Medicine, Kyoto, 602-8566 Japan; 4 Department of Molecular Cell Physiology, Graduate School of Medical Science, Kyoto Prefectural University of Medicine, Kyoto, 602-8566 Japan; 5 Japan Science and Technology Agency, PRESTO, Kawaguchi, Saitama, 332-0012 Japan; 6 Japan Science and Technology Agency, CREST, Kawaguchi, Saitama, 332-0012 Japan; 7 Perelman School of Medicine, University of Pennsylvania, Philadelphia, PA 19104 USA

**Keywords:** Autophagy, High-throughput screening, Protein design

## Abstract

Nonalcoholic steatohepatitis (NASH) is a progressive disorder with aberrant lipid accumulation and subsequent inflammatory and profibrotic response. Therapeutic efforts at lipid reduction via increasing cytoplasmic lipolysis unfortunately worsens hepatitis due to toxicity of liberated fatty acid. An alternative approach could be lipid reduction through autophagic disposal, i.e., lipophagy. We engineered a synthetic adaptor protein to induce lipophagy, combining a lipid droplet-targeting signal with optimized LC3-interacting domain. Activating hepatocyte lipophagy in vivo strongly mitigated both steatosis and hepatitis in a diet-induced mouse NASH model. Mechanistically, activated lipophagy promoted the excretion of lipid from hepatocytes, thereby suppressing harmful intracellular accumulation of nonesterified fatty acid. A high-content compound screen identified alpelisib and digoxin, clinically-approved compounds, as effective activators of lipophagy. Administration of alpelisib or digoxin in vivo strongly inhibited the transition to steatohepatitis. These data thus identify lipophagy as a promising therapeutic approach to prevent NASH progression.

## Abstract (web summary)

Nonalcoholic steatohepatitis (NASH) starts with lipid droplet accumulation in the liver that eventually causes inflammation and fibrosis. Here, authors use lipophagy activators to limit the accumulation of lipids in the liver and show that this can prevent disease progression in a mouse model of nonalcoholic steatohepatitis.

## Introduction

Lifestyle changes have led to worldwide increases in nonalcoholic fatty liver disease (NAFLD), with a prevalence now estimated to be around 25%^1^. Twenty to thirty percent of patients with NAFLD progress to nonalcoholic steatohepatitis (NASH), which in turn can progress to liver cirrhosis and eventually hepatocellular carcinoma. NASH has become the leading cause of liver failure in the US^2^. The pathogenesis of NASH development is complicated, involving multiple hits^3^. Excessive accumulation of lipid and abnormal fatty acid metabolism generate reactive oxygen species and endoplasmic reticulum (ER) stress. Infiltration by inflammatory cells and the subsequent fibrotic response drive NASH progression and contribute to increased mortality^4^. Circulating inflammatory monocytes are recruited via chemokine receptors^5^ and differentiate into liver macrophages, followed by activation of hepatic stellate cells (HSCs) and accumulation of extracellular matrix in the liver^6^. Despite the high prevalence and clinical importance of NAFLD, there is no approved drug for the treatment of NAFLD/NASH^7,8^.

Aberrant accumulation of lipid droplets (LDs) is the initial step of NAFLD/NASH pathogenesis, and it is thought that NASH is almost never resolved without improvement in steatosis^9^. However, dysregulated breakdown of LDs, despite lowering LD content, can contribute to disease progression. For example, studies with ablation of Perilipin 5, which normally prevents adipose triglyceride lipase (ATGL) transposition to LDs and thus prevents lipolysis^10^, show that activated lipolysis reduces hepatosteatosis but worsens hepatic lipotoxicity and insulin resistance^11,12^. Mechanistically, it is thought that the altered fatty acid composition caused by inefficient incorporation of fatty acids into LD-containing triacylglycerol drives NAFLD^13^.

Lysosomes provide an alternative pathway to degrade LDs, as well as other intracellular organelles and molecules, via various lysosomal hydrolases such as lipase, proteases, glycosidases, and nucleotidases^14^. Lysosomal acid lipase, LIPA (also known as LAL) degrades lysosomal lipids, and mutations in LIPA cause accumulation of LDs in various organs^15,16^, demonstrating the importance of lipophagy for lipid homeostasis. Lipophagy was first demonstrated in hepatocytes under nutrient deprivation^17^, but accumulating evidence shows that lipophagy contributes to LD degradation in numerous cell types, and that aberrant lipophagy is common in numerous diseases, including fatty liver, obesity, and cancers^18^. However, the molecular mechanism of how autophagy targets LDs remains poorly understood, with most mechanistic insight extrapolated from studies of bulk autophagy. In the liver, models of hepatocyte-specific autophagy deficiency have yielded complicated results, in part due to the lipogenic aspect of autophagosomes. For example, deletion of Atg7 or FIP200 impaired de novo lipogenic program by liver X receptor and protected from high-fat diet (HFD)-induced fat accumulation^4,19–22^. In contrast, activation of autophagy via overexpression of the transcription factor EB (TFEB) or by ablation of Rubicon clearly demonstrated the protective role of liver autophagy on NASH^23,24^. However, the specific role of lipophagy in these studies was not fully explored.

To definitely and specifically evaluate the effect of LDs disposal by autophagy, we developed here synthetic adapter proteins, fusing a LD-targeting signal (LDTS) with an optimized LC3 interacting region (LIR), to induce selective lipophagy of LDs and evaluate its impact on liver lipid homeostasis. Our adapter protein efficiently activated lipophagy and protected against NFALD and NASH in mice fed HFD or choline-deficient, L-amino acid-defined, high-fat diet (CDAHFD). Mechanistically, it was indicated that upregulated lipophagy may be coupled with lysosomal exocytosis, rather than fatty acid catabolism, to ultimately reduce liver fatty acid content and consequential inflammation and fibrosis. Finally, high throughput compound screening for lipophagy activators identified FDA-approved drugs that provide proof-of-concept for the potential clinical application of our findings.

## Results

### Development of an optimized lipophagy adapter protein

**Fig. 1:**
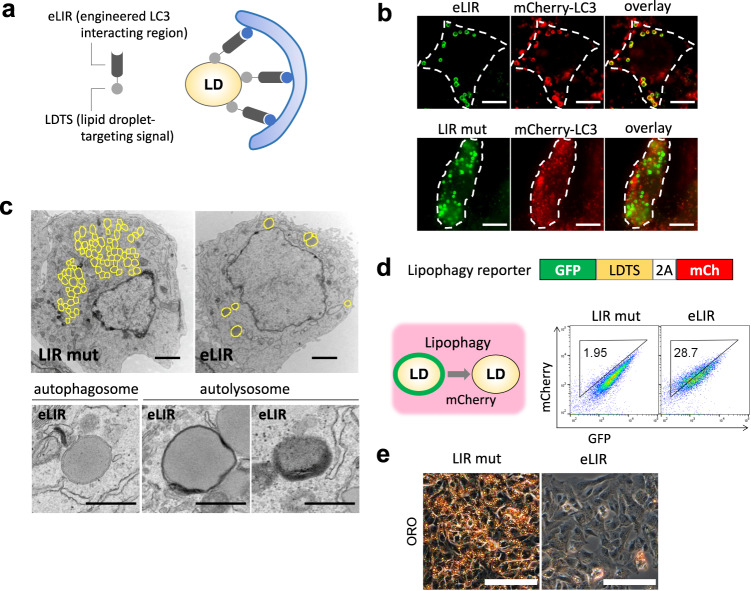
Synthetic adapter protein to induce lipophagy. **a** Schema of lipophagy-inducing synthetic adapter protein. The lipid droplet-targeting sequence (LDTS) from PLIN1 was fused with autophagosome recruiting engineered LC3 interacting region (eLIR). **b** 3T3L1 adipocytes expressing LDTS-mutant LIR (LIR mut) or LDTS-eLIR-GFP and mCherry-LC3 were analyzed by fluorescence microscopy. Scale bars, 10 µm. **c** HepG2 cells expressing LDTS-LIR mut or LDTS-eLIR were analyzed by electron microscopy. Lipid droplet is highlighted in yellow. Scale bars, 2 µm and 500 nm. **d** Lipophagy was quantified by flow cytometry for LDTS-GFP and internal control of mCherry in HepG2 cells expressing LDTS-LIR mut or LDTS-eLIR treated with oleic acid (OA) for 24 h. **e** HepG2 cells expressing LDTS-LIR mut or LDTS-eLIR were treated with OA for 24 h, and lipid droplets were stained with oil red O. Scale bars, 100 µm. These experiments were repeated independently 3 times with similar results (**b**, **c**, **e**).

We engineered a lipophagy adapter protein, designed to induce selective autophagy of LDs, by fusing a LDTS with an autophagosome-recruiting domain, i.e., an engineered LIR (eLIR) (Fig. 1a). To identify the effectual protein that best recruits autophagosome, we employed an established assay system detecting mitochondria-selective autophagy (mitophagy)^25^. First, ubiquitin-binding adapters and other LIR-containing proteins were fused with a mitochondrial outermembrane-targeting signal, and the level of mitophagy was analyzed with flow cytometry and the pH-indicator, mKeima^26^, which loses green fluorescence when in the presence of lysosomal low pH. This high-sensitive screening revealed that optineurin (OPTN) had the highest power to recruit autophagosome when expressed on the surface of mitochondria, and seemed more efficient than the previously reported AMBRA1-based mitophagy adapter protein^27^ (Supplementary Fig. 1a). OPTN is multi-functional protein modulating a number of signaling pathways like NF-κB, as well as trafficking of vesicles and proteins^28^. To prevent unintended effects of full-length OPTN expression, non-essential sequences were excluded, and residues 120–190 were identified as the minimal sequence necessary to promote autophagic degradation (Supplementary Fig. 1b). It is known that TBK1 phosphorylates serine residues near the LIR of ubiquitin-binding adapter proteins, including OPTN, to enhance binding affinity toward LC3 via conformational alteration and subsequent avidity effect^29^. Large aromatic side chains in the first residue of the LIR motif are also associated with higher affinity for LC3, and the affinity of the LIR in OPTN is promoted by replacing phenylalanine with tryptophan^30^. We therefore adopted two approaches to increasing LIR avidity: phosphomimetic mutation in 5 serine residues and tryptophan substitution in the core LIR motif. The resulting LIR exhibited more efficient recruitment of autophagosome in the context of free adapter protein modified from SunTag system^31^ and this configuration was therefore adopted as our eLIR of choice. As control, mutant LIR (LIR mut) with abolished affinity was designed by alanine substitution in 5 serine residues and the core LIR motif.

Next, we optimized the LDTS, using flow cytometry-based lipophagy assays in 3T3-L1 adipocytes. mKeima was fused with the LD surface protein, perilipin 1 to localize mKeima to LDs. Lipophagy was clearly induced in constructs fusing the eLIR with perilipin 1, but little with LDTS from hepatitis C virus^32^ or PNPLA5 (Supplementary Fig. 2a)^33^. The core domain of perilipin 1, which contains the LDTS^34^, was sufficient to induce lipophagy as efficiently as the full-length construct, and was more efficient than the LDTS from perilipin 5 (Supplementary Fig. 2b). Fluorescent imaging confirmed that the LDTS-eLIR was present on the surface of LDs and colocalized with LC3 labeled with mCherry (Fig. 1b), indicating targeting of LDs to the autophagosome. Electron micrographs exhibited LDs in autophagosome and autolysosome and the number of LDs was decreased in HepG2 hepatocytes expressing LDTS-eLIR (Fig. 1c). Lipophagy was also evaluated with administration of oleic acid to lipophagy-reporter HepG2 hepatocytes that express GFP-perilipin 1 and mCherry as a lipophagy reporter: when lipophagy occurs, the LD-associated GFP loses fluorescence in lysosomal acid conditions, while the mCherry signal is retained. Expression of the LDTS-eLIR construct strikingly activated lipophagy in these cells (Fig. 1d) and reduced the accumulation of LDs, as seen by staining with oil red O (Fig. 1e) or BODIPY (Supplementary Fig. 3a), or by flow cytometry (Supplementary Fig. 3b). The reduction of lipid content by LDTS-eLIR remained in ATGL knockout, which confirmed that lipid-lowering effect was independent of cytoplasmic lipase (Supplementary Fig. 3c). Our LDTS-eLIR construct activated lipophagy more reliably and selectively than did TFEB overexpression^23^ or starvation (Supplementary Fig. 3d). When compared with control vector, LDTS exhibited mild lipogenic effect (Supplementary Fig. 3b). Thus, we employed LDTS-LIR mut as a negative control to evaluate the effect of lipophagy activation with the LDTS-eLIR construct.

### Activation of hepatocyte lipophagy reverses steatohepatitis

**Fig. 2:**
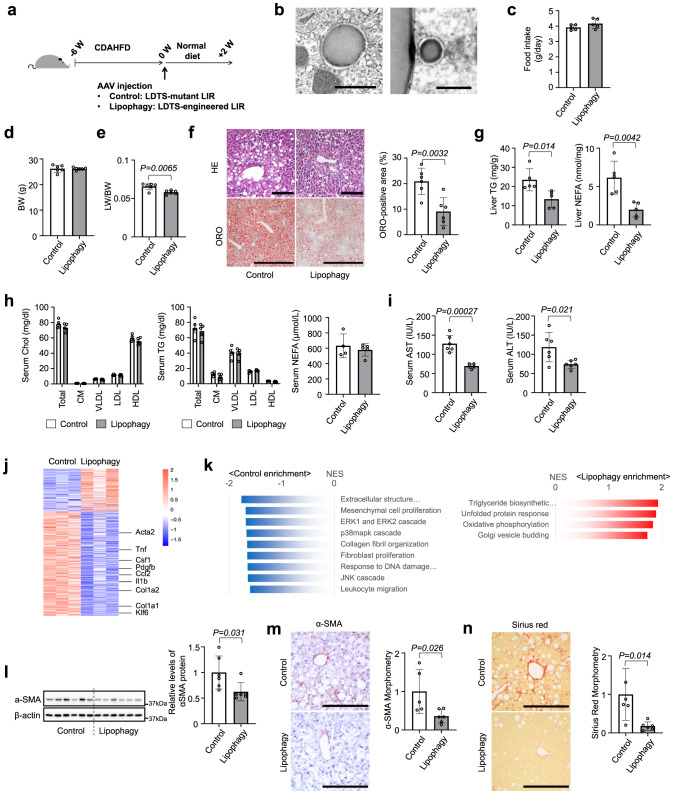
Lipophagy improved nonalcoholic steatohepatitis in mice fed CDAHFD. **a** Study protocol of NASH model with choline-deficient, L-amino acid-defined, high-fat diet (CDAHFD) and the induction of lipophagy with AAV-mediated gene delivery into hepatocytes. **b** Electron micrographs of lipophagy-induced liver tissues. Scale bars, 1 µm. **c**–**e** Food intake (**c**), body weight (**d**) and liver/body weight (LW/BW) ratios (**e**) of lipophagy-induced NASH model mice. **f** Hematoxylin/eosin or oil red O (ORO) staining of liver sections. Scale bars, 1 mm. **g** Liver TG and NEFA content. **h** Serum cholesterol and triglyceride (TG) in 4 major fractions and NEFA contents. **i** Serum AST and ALT levels. **j**, **k** RNA sequence and gene set enrichment analysis of liver tissues. **l** Immunoblots assessing the expression of αSMA in liver tissues. **m**, **n** Immunohistochemistry of αSMA (**m**) and sirius red staining (**n**) of liver sections and morphometry analysis of signal positive area. Scale bars, 200 µm. All data are presented as mean ± SD (*n* = 6 per group). *P* values calculated by two-sided unpaired *t*-test. Source data are provided as a Source data file.

To test the impact of activating lipophagy on NAFLD/NASH pathology, the LDTS-eLIR construct was engineered into serotype 8 adeno-associated virus (AAV8) with liver-specific thyroxine-binding globulin promoter (pTBG). Consistent with lipogenic effects of LDTS in vitro, 4-week expression of LDTS induced lipid accumulation in the liver (Supplementary Fig. 3e). Therefore, AAV8s carrying LDTS-eLIR and LDTS- LIR mut were injected in mice and compared after 6-weeks of feeding CDAHFD as a NASH model^35^. Mice were then switched to normal chow, and analyzed 2-week later (Fig. 2a). Electron micrographs of liver tissue demonstrated that the occurrence of lipophagy in LDTS-eLIR-induced mouse liver tissue (Fig. 2b). The introduction of LDTS-eLIR did not affect food intake or body weight, but it did reduce liver weight significantly (Fig. 2c–e). This was accompanied by markedly decreased liver steatosis, as evidenced by H&E histology, oil red O staining, and quantification of triglyceride and nonesterified fatty acid content (Fig. 2f, g). Importantly, serum lipid profile remained unaltered in animals treated with LDTS-eLIR (Fig. 2h). The reduced steatosis in the liver was associated with evidence of mitigated liver injury (Fig. 2i). Gene set enrichment analysis of RNAseq of liver tissue indicated that activation of lipophagy mitigated profibrotic and proinflammatory responses (Fig. 2j, k). Consistent with this, α-smooth muscle actin (α-SMA) protein expression, a marker of HSC activation, and fibrosis staining (Sirius red) were strongly attenuated in the lipophagy-induced liver (Fig. 2l–n). The induction of lipophagy also led to higher expression of mitochondrial oxidative phosphorylation (OXPHOS) genes that might reflect milder NASH pathology (37) (Fig. 2k).

To test the prediction that the protection from NASH conferred by LDTS-eLIR requires the autophagy machinery, the hepatoprotective effect of LDTS-eLIR was assessed in autophagy-deficient settings of liver-specific *Atg5* knockout mice or chloroquine (CQ)-treated mice. *Atg5* gene deletion was achieved by two AAV vectors for concurrent gene delivery of Cre recombinase with LDTS-LIRs by the AAV8 pTBG system (Supplementary Fig. 4a, b). In the absence of liver Atg5, the expression of LDTS-eLIR protein induced little alteration of LDs, liver triglycerides, and nonesterified fatty acids (Supplementary Fig. 4c, d). CQ is a classic inhibitor of autophagy by disturbing autophagosome fusion with lysosomes^36^. Like deletion of ATG5, treatment with CQ also prevented the lipid-lowering effect of LDTS-eLIR in HepG2 cells (Supplementary Fig. 4e) and livers from mice fed CDAHFD (Supplementary Fig. 4f–h). These results thus indicate that both autophagosome formation and subsequent fusion with lysosome are essential for the hepatoprotective effect of LDTS-eLIR.

**Fig. 3:**
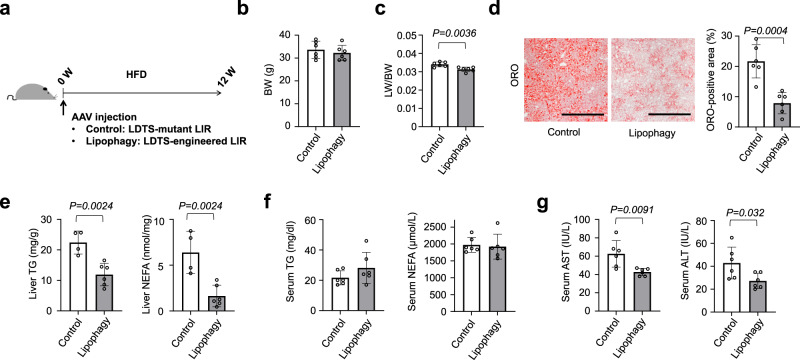
Lipophagy prevented hepatosteatosis in mice fed HFD. **a** Study protocol of hepatosteatosis model with high-fat diet (HFD) and the induction of lipophagy with AAV-mediated gene delivery into hepatocytes. **b**, **c** Body weight (**b**) and liver/body weight (LW/BW) ratios (**c**) of lipophagy-induced mice fed HFD. **d** ORO staining of liver sections. Scale bars, 1 mm. **e**, **f** The levels of TG and NEFA in the liver (**e**) and serum (**f**). **g** Serum AST and ALT levels. All data are presented as mean ± SD (*n* = 6 per group). *P* values calculated by two-sided unpaired *t*-test. Source data are provided as a Source data file.

We also examined the protective effect of lipophagy on liver steatosis in a standard HFD model. Lipophagy was similarly induced with AAV-mediated gene delivery and mice were fed HFD for 12 weeks (Fig. 3a). Lipophagy did not alter body weight but reduced liver weight (Fig. 3b, c). Liver staining with oil red O showed attenuated lipid accumulation with induction of lipophagy, and biochemical analyses confirmed lower triglyceride and nonesterified fatty acid in lipophagy-induced livers without affecting serum profiles (Fig. 3d–f). Even though HFD induced modest liver injury as compared with CDAHFD, lipophagy reduced the levels of serum AST and ALT (Fig. 3g).

### Activation of lipophagy may promote lysosomal exocytosis of lipids

**Fig. 4:**
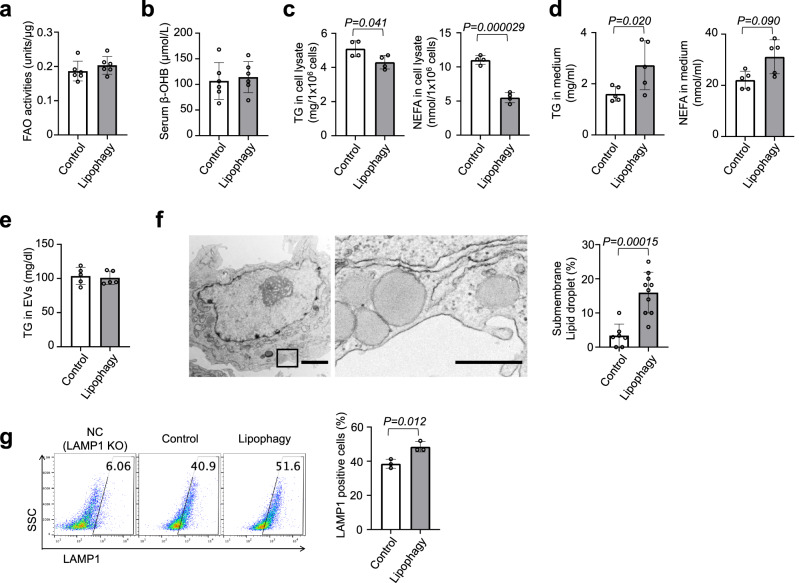
Lipophagy excreted LDs through the lysosomal exocytosis. **a** Fatty acid oxidation (FAO) activities of liver tissues in mice fed CDAHFD for the NASH model. **b** The levels of serum ß-OHB in mice fed CDAHFD (**a**, **b**; *n* = 6 per group). **c**, **d** HepG2 cells expressing LDTS-LIR mut (control) or eLIR (lipophagy) were cultured in medium containing oleic and palmitic acid (OA + PA) for 12 h and then incubated in FBS-free medium for 6 h. TG and NEFA of cell lysate (**c**) and culture supernatant (**d**) were measured (*n* = 4 per group). **e** TG content in serum exosomes in a mouse model of NASH fed with CDAHFD (*n* = 5 per group). **f** Electron micrographs of lipophagy-induced hepatocyte. Right image is magnified view of left black square. LDs localized close to cellular membrane was quantified. Scale bars, 2 µm and 500 nm (*n* = 10 cells per group). **g** Flow cytometry for Lamp1 in the surface of HepG2 cells expressing LDTS-LIR mut (control) or eLIR (lipophagy). LAMP1 KO cells were used as a negative control (*n* = 3 per group). All data are presented as mean ± SD. *P* values calculated by two-sided unpaired *t*-test. ß-OHB; ß-Hydroxybutyric acid. Source data are provided as a Source data file.

To investigate the fate of lipids removed by lipophagy, we first assessed lipid catabolism in livers with LDTS-eLIR. Lipophagy-induced liver tissue showed no overall significant alteration of transcription of fatty acid oxidation (FAO), lipolysis, or lipid biosynthesis genes, despite the higher expression of mitochondrial respiration-related genes (Supplementary Fig. 5a, b). Functionally, there was no difference in FAO enzymatic activity of liver lysates, nor in the serum level of β-OHB, a marker of excess fatty acid oxidation in the liver (Fig. 4a, b). FAO activity in cultured HepG2 cells, analyzed using a quenched fluorescent probe that is activated by the sequential enzyme reactions of FAO^37^, also revealed no changes in FAO in lipophagy-activated cells (Supplementary Fig. 5c). Oxidation thus seems unlikely to be the ultimate fate of lipids removed by lipophagy.

While cellular triglyceride and nonesterified fatty acid were decreased upon induction of lipophagy in HepG2 cells treated with oleic and palmitic acid (Fig. 4c), levels of triglyceride in cultured medium were increased (Fig. 4d). We thus hypothesized that activation of lipophagy caused the induction of lipid export, thereby contributing to liver protection. In mice fed CDAHFD, lipophagy did not change the amount of triglyceride in extracellular vesicles (EVs) (Fig. 4e), suggesting alternative pathways of secretion. Electron micrograph of lipophagy-induced hepatocytes showed that LDs were localized close to the cell membrane (Fig. 4f). In light of these observations, and essentiality of autophagosome fusion with lysosomes in the protective effect of LDTS-eLIR in CQ-treated mice experiments (Supplementary Fig. 4f–h), we focused our attention on lysosomal exocytosis.

**Fig. 5:**
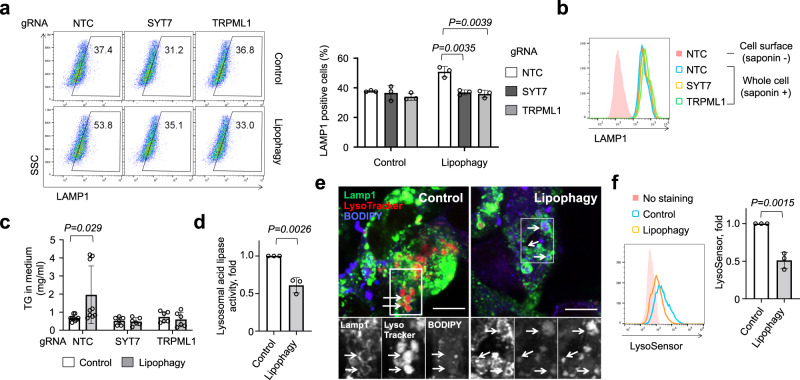
Deacidification activated lysosomal exocytosis. **a** Flow cytometry for Lamp1 in the surface of control and lipophagy-induced HepG2 cells. SYT7 or TRPML1 was knockout by CRISPR-Cas9 (*n* = 3 per group). **b** Flow cytometry to evaluate whole cell expression level of LAMP1 in saponin-treated cells. **c** TG in the medium of control and lipophagy-induced HepG2 cells. Cells were cultured with FFAs (OA + PA) containing medium for 12 h and then maintained in FBS-free medium for 6 h (*n* = 9 per NTC group, *n* = 6 per SYT7 and TRPML1 group). **d** Lysosome acid lipase activity in control and lipophagy-induced HepG2 cells (*n* = 3 per group). **e** Lamp1, LysoTracker Red DND-99 and BODIPY co-staining in control and lipophagy-induced HepG2 cells. Scale bar, 10 mm. **f** Flow cytometry for LysoSensor Green DND-189 staining of control and lipophagy-induced HepG2 cells (*n* = 3 per group). All data are presented as mean ± SD. *P* values calculated by two-sided unpaired *t*-test. Source data are provided as a Source data file.

Lysosomal exocytosis causes the accumulation of LAMP1 at the cell surface^38^, and introduction of LDTS-eLIR in HepG2 cells significantly increased the amount of cell surface LAMP1 (Fig. 4g). Interestingly, this evidence of lysosomal exocytosis was not observed after activation of other forms of selective autophagy, such as mitophagy and pexophagy (the latter induced by fusion protein of the localization motif of the peroxin Pex13^39^ and eLIR, and monitored by peroxisome-localized mKeima with the carboxyl-terminal amino acid sequence serine-lysine-leucine^40^) (Supplementary Fig. 6a, b). To better understand the mechanism underlying the activation of lysosomal exocytosis by lipophagy, we focused on the Ca^2+^ signal-dependent lysosomal fusion with cell membrane^41,42^. Knockout of the lysosome Ca^2+^ channel, TRPML1, or the cell membrane Ca^2+^ sensor, SYT7, completely blocked the induction of cell surface LAMP1 by the activation of lipophagy (Fig. 5a, b). Similarly, lipophagy-mediated excretion of triglyceride was completely blocked in *SYT7* or *TRPML1* knockout cells (Fig. 5c). We conclude that activation of lipophagy leads to Ca^2+^-dependent lysosomal exocytosis of triglycerides.

Inhibition of mTORC1 is one of the signals for TRPML1-mediated Ca^2+^ efflux^43^, but no alteration of mTORC1 activity was observed after activation of lipophagy (Supplementary Fig. 6c). Next, we examined the contribution of fatty acids to Ca^2+^ signal in cells expressing cell surface-localized mutant TRPML1 channels^44^. Monitoring Fura-2 revealed that neither oleic acid nor fatty acid mixture stimulated TRPML1 to increase cytosolic Ca^2+^ (Supplementary Fig. 6d). In considering other possible triggers for lysosomal exocytosis, we noted that, in the assay for mKeima-based lipophagy, the shift of acidic signal was small as compared with the case of mitophagy (Supplementary Figs. 1 and 2). The GFP-based assay also exhibited relatively smaller signal alteration, and additional starvation, known to activate lysosomal acidification^45^, increased the signal (Supplementary Fig. 3d). These observations suggested a defect in lysosomal acidification upon induction of lipophagy. Lysosomal enzymes function in acidic pH, and even small increases in pH impair enzyme activity^46^. Consistent with this, lysosomal lipase activity, measured with self-quenched lipase substrates^47^, was reduced in lipophagy-induced cells (Fig. 5d). Staining with a cell-permeable and acidotropic LysoTracker Red DND-99 showed that lipid-containing lysosome had decreased signal in lipophagy-induced cells (Fig. 5e), and lipophagy reduced fluorescence by LysoSensor, a dye sensitive to pH alteration (Fig. 5f). These results thus indicate that lipophagy disturbs lysosomal acidification, likely consequently stimulating Ca^2+^ release through TRP channel^48^ and subsequent lysosomal exocytosis.

Finally, we noted that if activation of liver lipophagy promotes secretion of lipids, a concern can be raised of ectopic lipid accumulation and lipotoxicity. However, we observed no hypertrophy of white adipose tissue and no lipid accumulation in skeletal muscle and kidney (Supplementary Fig. 7a–e). Similarly, glucose and insulin profiles showed no significant differences upon activation of liver lipophagy in animals subjected to a CDAHFD (Supplementary Fig. 7f–i).

### High-throughput compound screen identified lipophagy activators that suppress NASH progression

**Fig. 6:**
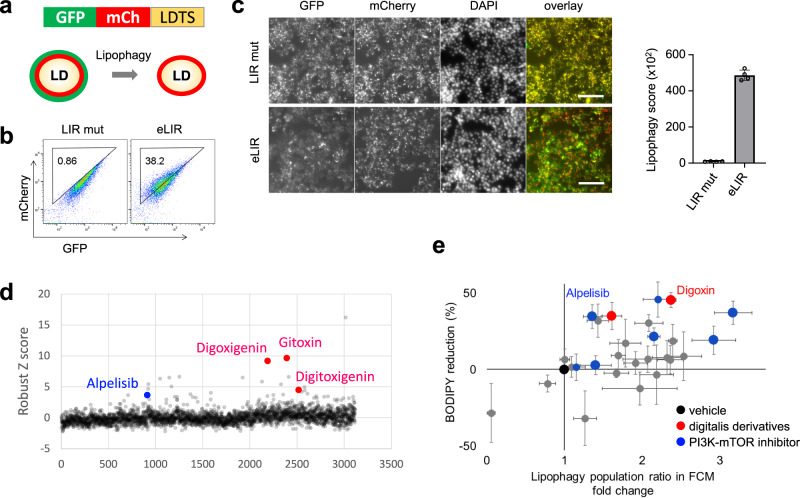
High content compound screening to find lipophagy activators. **a** Schematic diagram showing the image-based reporter system to quantify lipophagy activity level. GFP loses its fluorescence in autolysosomes resulting in a decrease in total fluorescent intensity of GFP in mCherry positive area. **b** The quality of lipophagy reporter system was confirmed by flow cytometry with LDTS-LIRs. **c** Automated imaging of lipophagy reporter cells expressing LDTS-LIRs as controls. Scale bars, 100 µm (*n* = 4 per group). **d** Robust Z score of approximately 3500 small molecules library. Toxic compounds defined as cell number <80% were excluded. **e** Individual validation of lipophagy and assessment of intracellular lipid lowering effect among 23 out of 39 primary hits, digoxin and other PI3K-mTOR inhibitors. All data are presented as mean ± SD. *P* values calculated by two-sided unpaired *t*-test. Source data are provided as a Source data file.

We performed a small-molecule screen to identify activators of lipophagy, with an eye to developing lipophagy-based NASH therapy. HepG2 hepatocytes stably expressing tandem GFP-mCherry-LDTS were generated for image-based screening of lipophagy (Fig. 6a). Normal LDs are observed as GFP and mCherry double positive dots, whereas LDs undergoing lipophagy lose GFP signal and become mCherry single positive dots. As a positive control, transduction of LDTS-eLIR strikingly increased lipophagy in this assay (Fig. 6b). In the imaging analyses, lipophagy score was defined as a subtraction of total fluorescence amount of GFP from that of mCherry in mCherry positive area per cell. LDTS-eLIR expression exhibited about 50 folds-increase in lipophagy score in this analysis (Fig. 6c), consistent with the result of flow cytometry (Fig. 6b). We next screened approximately 3500 small molecules, including 1600 clinically-approved drugs and 1900 validated compounds provided by Drug Discovery Initiative in The University of Tokyo. Among 39 primary hits defined as 3< robust Z score, there were three digitalis derivatives, three mTOR pathway inhibitors, and one PI3K inhibitor (Fig. 6d and Supplementary Data 1). Twenty-three out of 39, digoxin and other PI3K-mTOR inhibitors were validated and also tested for impact on lipid lowering under oleic acid containing culture conditions (Fig. 6e).

**Fig. 7:**
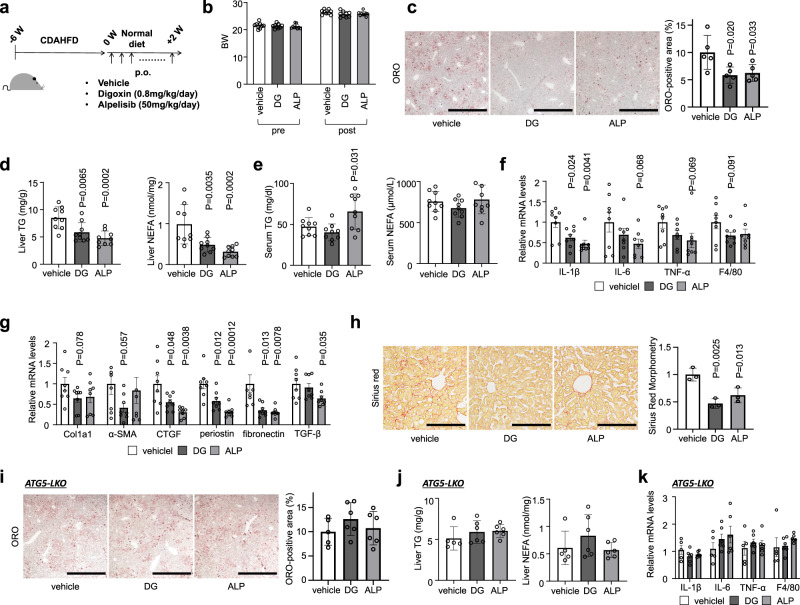
Alpelisib and digoxin prevented the progression of NASH in mice fed CDAHFD. **a** Study protocol of digoxin (DG) or alpelisib (ALP) administration in NASH model mice with CDAHFD. **b** Body weight of control and drugtreated mice. **c** ORO staining of liver sections. Scale bars, 1 mm. **d** Liver TG and NEFA content. **e** Serum TG and NEFA levels. **f**, **g** Real-time PCR assessing the expression of inflammation- (**f**) and fibrosis- (**g**) related genes in liver tissue. **h** Sirius red staining of liver sections. Scale bars, 200 µm. **i** ORO staining of liver sections of liver-specific *Atg5* knockout mice fed CDAHFD. **j** Liver TG and NEFA content of liver-specific *Atg5* knockout mice fed CDAHFD. **k** Real-time PCR assessing the expression of inflammation-related genes in *Atg5* knockout liver tissue. Data are presented as mean ± SD of *n* = 8–9 (**a**–**h**) and *n* = 5–6 (**i**–**k**). *P* values calculated by one-way ANOVA with Dunnett’s multiple comparison test. Source data are provided as a Source data file.

**Fig. 8:**
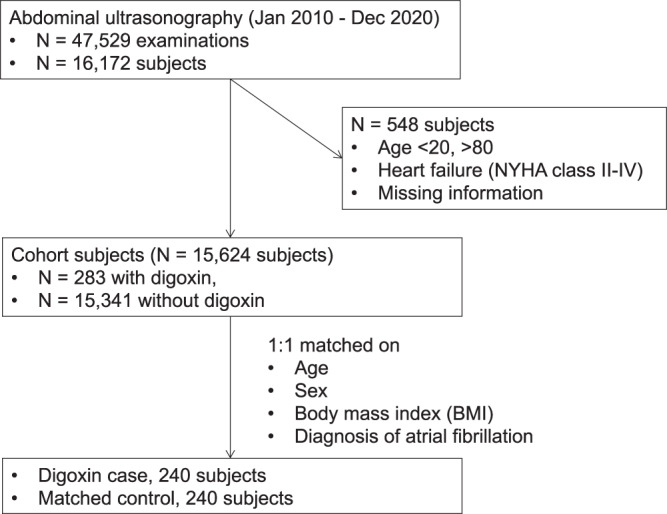
Flow diagram of selection in the nested case-control study. The results are provided in Table 1.

We chose two validated hit compounds for further in vivo studies: alpelisib and digoxin. Administered of these clinically-approved compounds to mice fed CDAHFD (Fig. 7a) had no impact on body weight but significantly reduced hepatic steatosis, as seen by oil red O staining, and quantified triglyceride and nonesterified fatty acid content (Fig. 7b–d). There were no alterations in serum lipid levels except mild triglyceride increase in alpelisib treatment (Fig. 7e). Alpelisib and digoxin both attenuated inflammatory and profibrotic gene expression, and suppressed liver fibrosis (Fig. 7f–h). Treatment of liver-specific *Atg5* knockout mice fed CDAHFD with either drug failed to elicit any reduction of liver lipid profiles (Fig. 7i, j), indicating that the protection afforded by alpelisib or digoxin treatment is indeed mediated by lipophagy. In addition, reduced inflammation observed in wild-type livers was not reproduced when *Atg5* was deleted (Fig. 7k). Alpelisib and digoxin had no anti-inflammatory activity and the hepatoprotective effect of these compounds was confirmed to be dependent on lipophagy,

Finally, we retrospectively examined the effect of digoxin administration on indices of fatty liver in human subjects. Digoxin is mainly used in the treatment of heart failure and atrial fibrillation, both of which are associated with NAFLD^49^. We conducted a nested case-control study, matching for age, gender, BMI, and atrial fibrillation, and excluding patients with heart failure (NYHA class II–IV) to remove the hemodynamic contribution of digoxin to livers. A total of 16,172 patients who underwent abdominal ultrasonography in the hospital of Kyoto Prefectural University of Medicine between 2011 and 2020 were included in this study. Among these subjects, 548 were excluded due to missing data, heart failure (NYHA class II-IV) or age more than 80 or less than 20 years old, leaving a total of 15,624 patients, of which 282 were actively receiving digoxin treatment. The case-control cohort was selected 1:1, 240 cases in each group (Fig. 8). Fatty liver was diagnosed by hepatorenal echo contrast and liver brightness on abdominal ultrasonography^50^. Strikingly, the prevalence of fatty liver was significantly lower in subjects receiving digoxin than those not receiving digoxin (Table 1).

**Table 1:** Prevalence of fatty liver subjects with Digitalis preparation and those without

	Control	Digoxin	*P* value
Number of subjects	240	240	
Fatty liver	23.8% (57)	15.4% (37)	0.025
Atrial fibrillation	75.0% (180)	75.0% (180)	
Male	62.5% (150)	62.5% (150)	
Age, years	71.3 ± 9.6	71.3 ± 9.6	0.96
BMI, kg/m^2^	21.3 ± 3.1	21.3 ± 3.1	0.88

Continuous variables are expressed as mean ± SD and are compared by two-sided unpaired *t*-test. Categorical variables are expressed as % and number, and are compared by Mantel-Haenzel test.

## Discussion

We show here that liver LDs can be safely and efficiently eliminated by lipophagy, which processes lipids to extrahepatic secretion rather than to catabolism by mitochondrial FAO, and ameliorates steatosis without inflammatory or fibrotic liver injuries. More generally, our synthetic lipophagy adapter protein, comprised of a LD-localizing sequence fused to the optimized LIR, enables the selective analysis of LD autophagy, in contrast to bulk autophagy.

NAFLD begins with excessive lipid accumulation in the liver and, in some cases, progresses to NASH and liver cirrhosis^13^. Hepatosteatosis is mainly caused by overnutrition, obesity, and insulin resistance. Other mechanisms subsequently contribute to liver injury, including oxidative stress, ER stress, mitochondrial dysfunction, as well as immunological alterations. The complex pathogenesis of NAFLD/NASH underscores the potential benefit of intervening in the initial steps of the disease, i.e., lipid accumulation. Lipid accumulation reflects an imbalance between lipogenesis and lipolysis. Inhibiting lipogenesis can reduce steatosis without increasing inflammatory and profibrotic response, as seen for example in mice lacking hepatic Diacylglycerol Acyltransferase 2 (DGAT2)^51,52^. Conversely, cytoplasmic lipolysis also decreases steatosis; however, in this case the ensuing production of cytoplasmic nonesterified fatty acids induces hepatic inflammation and systemic insulin resistance^11,12^. In contrast, the present study demonstrates that the lipolysis of LDs in autophagosomes and lysosomes via lipophagy safely disposes the lipid to the outside the liver and protects from lipotoxicity.

Previous efforts to promote liver lipophagy involved manipulating bulk autophagy. However, the LD autophagy was only a small fraction of total autophagy, even when maximally stimulated by starvation (Supplementary Fig. 3d). Our study is the first to examine the effect specifically of lipophagy on NAFLD/NASH pathology. To induce selective autophagy, we generated an adapter protein composed of an LD-targeting sequence and an autophagosome recruiting domain. Similar adapter approaches to achieve selective autophagy were previously reported using p62^53^, AMBRA1^27^, or small compounds^54,55^ as LIR. We employed instead OPTN, which we found to have stronger affinity to autophagosome, and additionally introduced mutations to enhance the affinity against LC3. In addition, OPTN was trimmed to the minimal required sequence, to avoid unexpected effects mediated by other protein-protein interactions, as commonly seen with LC3 receptors^28^. Thus, our newly engineered LIR is short, has strong capacity for autophagosome recruitment, and carries minimal risk of other effects. Our optimized approach can be extended to other selective autophagies with proper targeting domains, including mitophagy and pexophagy as shown here as well.

The canonical fate of autophagy is the degradation of cargo by lysosomal enzymes. Alternatively, autophagy can also have a secretory fate through exocytosis of autophagosomes or autolysosomes^56^. The mechanisms underlying this fate decision remain largely unknown. Recent work showed that autophagosome-derived vesicles mainly contained RNA binding proteins, which controlled their secretion by interacting with LC3 and others^57^. In the case of lipophagy, we found lipid content to be altered significantly in cultured medium, but not in isolated EVs, which can be derived from late-endosome and autophagosome, suggesting an alternative pathway of lipid exocytosis^58^. The lipophagy-mediated improvement of steatosis was abrogated by inhibition of the fusion of autophagosome and lysosome, and the cell surface expression of the lysosomal protein LAMP1 was upregulated by lipophagy, a common marker of lysosomal exocytosis^59^. These results collectively suggest that lysosomal exocytosis, rather than secretory autophagy, was responsible for lipophagy-mediated lipid secretion.

Lysosome secretion involves the migration of lysosomes from the perinuclear region to the cell surface and fusion with the cell membrane. It plays an important role in bone resorption, antigen presentation, and cell membrane repair^60^. Lysosomal fusion with the cell membrane is mediated by Ca^2+^ signaling. Lysosomes store Ca^2+^ to a concentration similar to that in endoplasmic reticulum (ER)^61^. Lysosomal Ca^2+^ is released through the TRPML1 Ca^2+^ channel, and the secreted Ca^2+^ is sensed by cell membrane-associated SYT7, which activates the SNARE complex to promote lipid bilayer fusion of lysosomes and plasma membrane^62^. Starvation induces TRPML1-mediated Ca^2+^ efflux, possibly through mTOR inhibition^43^, and a recent study reported that starvation-mediated autophagy of LDs exported fatty acid through lysosomal exocytosis, which required lipolysis of the TG cargo to activate the exocytosis^63^. However, in our study, lipophagy was not accompanied by mTOR inhibition (Supplementary Fig. 6c); our Ca^2+^ assay suggested that fatty acids did not change the Ca^2+^ dynamics (Supplementary Fig. 6d); the lipid cargo was exported without lipase-mediated catabolization to nonesterified fatty acid (Fig. 3d); and lysosomal exocytosis was not activated by selective autophagy of peroxisome, despite their high content of fatty acids (Supplementary Fig. 6b). Thus, triglycerides, rather than fatty acids, might be involved in lysosomal deacidification and subsequent TRPML1-mediated Ca^2+^ efflux and shifting lysosome to exocytosis. One possibility is that loading too much cargo disturbs the trafficking of proton pump or ion channels, but the detailed mechanism of deacidification requires further investigation.

Accumulating evidence indicates that cellular vesicle dynamics are complicated, likely explaining why the deletion of *Atg5* did not fully block the effect of LDTS-eLIR. For example, LC3-positive endosome or multivesicular body were recently identified by several groups^57,64,65^. Our adapter protein is predicted to recruit these LC3-positive vesicles in addition to conventional autophagosome, which could result in ATG5-independent lysosomal exocytosis. In addition, *Atg5* knockout could compensatively activates alternative vesicle dynamics and LIR-LC3-dependent processing of LDs, leading to further relative reduction of liver lipid content by LDTS-eLIR.

To identify potential inducers of lipophagy to treat NAFLD/NASH, we performed a small molecule library screen, using a newly developed lipophagy reporter system. Approximately 3500 clinically approved or functionally validated compounds were screened and several digitalis derivatives and PI3K-mTOR pathway inhibitors were identified as lipophagy activators. Among them, digoxin and a PI3K inhibitor, alpelisib were confirmed and showed significant therapeutic effects in vivo in a mouse model of NASH. Alpelisib is used to treat a PIK3CA-mutated, hormone receptor-positive, human epidermal growth factor receptor 2-negative breast cancer^66^. We treated mice at a dose of 50 mg/kg/day that was the same as chemotherapy regimen for pre-clinical murine models of osteosarcoma^67^ but higher than 300 mg/day for human breast cancer. Although we have limited information about side effects, we did not observe body weight loss, which was one of major side effects in clinical trials of alpelisib^66^ (Fig. 6b). Previous studies have reported the therapeutic potential of digoxin in NAFLD/NASH^68,69^. One study reported that digoxin binds pyruvate kinase M2 (PKM2) and inhibits chromatin remodeling, leading to downregulation of inflammatory HIF-1a transactivation^68^. The other study identified cardiac glycosides as TFEB activators, via the known function of digoxin as an inhibitor of ATP-dependent Na+-K+ transporter, consequently activating Ca^2+^ influx through the sodium-calcium exchanger or ER Ca^2+^ release by IP3R, in turn driving TFEB dephosphorylation and activation through calcineurin and an unknown phosphatase^69,70^. In our study, unbiased screening identified digoxin as a lipophagy inducer, and we demonstrated in vivo that the protective effect of digoxin on the liver was entirely dependent on autophagy. Digoxin has long been used clinically to modulate cardiac contractility and the conduction system. We took advantage of the prevalent use of digoxin to carry out a nested case-control study, in which we demonstrated significantly lower prevalence of fatty liver in patients prescribed digoxin. We conclude that digoxin provides a potential novel approach to protect against steatosis, although further clinical research is required.

There are limitations in the present sturdy. First, LDTS derived from perilipin 1 had in part the lipogenic effect. We thus evaluated the effect of LDTS-eLIR comparing with LDTS-LIR mut to equalize the background. AAV-mediated gene delivery of LDTS-eLIR demonstrated the hepatoprotective effect of lipophagy in NAFLD/NASH models. However, it is required to employ pure LDTS without affecting lipogenesis for clinical application of lipophagy adapter protein. Second, in associattion with LDTS lipogenic property, our NASH model was not ongoing CDAHFD. Deficient choline and limited methionine impair VLDL secretion and induce rapid and severe lipid accumulation in hepatocytes. It is a good model to replicate human NASH in highly-accelerated time course. However, we presume that its lipid accumulation was faster than lipophagy-mediated lipid export kinetics and the lipogenic effect of LDTS further accelerated liver steatosis. Therefore, lipophagy couldn’t show the protective effect in uninterrupted CDAHFD. Recently, another study reported the protective effect of lipophagy with the engineering of autophagy-tethering compound. This lipophagy-inducing compound had no lipogenic effect and reduced liver steatosis even in uninterrupted CDAHFD^55^. Our lipophagy was also clearly effective in the case of slower-paced lipid accumulation in HFD-induced NAFLD. Human NAFLD/NASH progresses in years, not in weeks. Thus, these results conclude that lipophagy has the potential to protect livers from NAFLD/NASH. Third, to rule out the lysosomal lipolysis-mitochondrial FAO pathway, we addressed no alteration of FAO-related transcription and enzymatic activity. Although serum level of β-OHB is a marker of excess fatty acid oxidation in the liver and exhibited no changes upon lipophagy activation, the quantification of beta oxidation of endogenous fatty acids using Seahorse extracellular flux analysis could further provide the evidence related to the fate of LDs. In addition, demonstration of LD markers in the cultured medium upon lipophagy could convincingly support the concept of LD exocytosis.

In summary, our study demonstrates that a selective autophagy-inducing adapter protein composed of a specific perilipin 1-derived LD-targeting domain fused to optimized LIR robustly activates lipophagy, safely disposes of aberrantly accumulated lipid, and protects the liver from NAFLD/NASH in a mouse model. The system was also leveraged to establish a high-throughput screening system to identify lipophagy regulators and identify two FDA-approved drugs as lipophagy activators. The work provides multi-faceted proof-of-concept for the potential clinical application of lipophagy-based therapies.

## Methods

### Mice

All mouse experiments were approved by the Animal Care and Use Committee of the Kyoto Prefectural University of Medicine (M2020-59). C57BL/6J mice were purchased from CLEA Japan, Inc. B6.129S-Atg5<tm1Myok> (C57/BL6J background) were provided by the RIKEN BRC through the National Bio-Resource Project of the MEXT/AMED, Japan^71^. Chronic liver injury was induced by feeding a choline-deficient, L-amino acid-defined, high-fat diet (CDAHFD; Research Diets Inc., A06071302) to 8-weeks-old male C57BL/6J or B6.129S-Atg5<tm1Myok> mice for 6 weeks. The CDAHFD mice were grouped so that the average body weights of mice in each group were similar. The grouped mice were intravenously injected with a 2E11 genome copies (GC)/mouse of lipophagy induced (LDTD-LIR) or control (LDTD-LIR mut) AAVs once, or orally administered digoxin (DG) (0.8 mg/kg/day, Toronto Research Chemicals Inc, D446575), alpelisib (ALP) (50 mg/kg/day, MedChem Express) or its solvent 0.5 w/v% Methyl Cellulose solution (WAKO) once daily for two weeks^67,69^. In some experiments, 65 mg/kg chloroquine (Sigma-Aldrich) was administered by daily intraperitoneal injections. For simple steatosis model, lipphagy was induced similarly by AAV injection and the mice were fed high-fat diet (HFD; Oriental Yeast, HFD-60) for 12 weeks. Mice were maintained in a specific pathogen-free animal facility on a 13:11 h light–dark cycle at an ambient temperature of 21 °C with 55% humidity. They were given free access to water and food. Age- and sex-matched mice were used for all animal experiments.

### Human study

The nested case-control study was performed in Kyoto Prefectural University of medicine. This cohort study was approved by Clinical Research Review Committee in Kyoto Prefectural University of medicine (CRB5200001, ERB-C-2104). Opt-out method was employed to obtain consent on this study. A description of the research and contact information was made available on the website. It was explained that they were free to opt out of participation in the study by phone or through the website. Following information was extracted from the electronic medical records of Kyoto Prefectural University of Medicine Hospital for patients who underwent abdominal ultrasonography between 2010/1/4 and 2020/12/28: age, sex, height, body weight, whether atrial fibrillation was diagnosed, and whether digoxin was administered. In cases with multiple abdominal ultrasound examinations, the date of the most recent abdominal ultrasound examination was included in the analysis. The inclusion criteria were 20 to 80 years of age, and the exclusion criteria were heart failure (NYHA class II–IV) and missing information. Fatty liver was diagnosed by the findings of abdominal ultrasonography performed by a trained technician. The parenchymal brightness with liver-to-kidney contrast was used as a diagnostic parameter, which allowed for reliable and accurate detection of fatty liver^72^. The subjects were selected 1:1 against case control with or without digoxin, with age, sex, BMI, and diagnosis for atrial fibrillation as matching criteria. The frequency of fatty liver in each group was examined by T-test. EZR (R version 4.0.3)^73^ was used for statistical analysis. The significance level was set at *P* < 0.05. As a result, information on 47,529 measurements of abdominal ultrasound was obtained from this period. The cohort consisted of 16,172 subjects, and 548 subjects were excluded due to heart failure (NYHA class II-IV) and missing information, leaving 15,624 subjects for analysis. In this cohort, there were 283 subjects who received digoxin. Nested case-control study consisted of 240 cases in each group, for a total of 480 cases.

### Cells

HepG2 cells, 3T3L1 cells and Lenti-X 293T cells were cultured at 37 °C with 5% CO_2_ in Dulbecco’s modified Eagle’s medium (DMEM, Invitrogen) containing 10% fetal bovine serum (HyClone) and penicillin/streptomycin (100 U/ml, Invitrogen). To establish an NAFLD model in HepG2 cells, 0.1 mM oleic acid (Sigma-Aldrich), or 0.5 mM FFAs (oleic acid/palmitic acid (Sigma-Aldrich), 2:1) for 24 h^74^. In some experiments, HepG2 cells were cultured with 25 µM chloroquine (Sigma-Aldrich) for 24 h or 5 nM Bafiromycin A1 (Sigma-Aldrich) for 2 h^75^. HepG2 cells and 3T3L1 cells were purchased from ATCC, and Lenti-X 293T cells were from Clontech. No commonly misidentified cell line was used in this study. All the cell lines were routinely tested negative for mycoplasma contamination.

### Plasmids

Individual gRNAs were cloned into lentiCRISPR v2 (Addgene 52961) or lentiGuide-Puro (Addgene 52963) and cDNAs were cloned in pLenti (Addgene #22255), pMSCV (Clontech), pAAV TBG (Adddgene #105535), or pcDNA4TO (Addgene #60914). Drug resistance genes in lentiGuide, pLenti, and pMSCV were replaced by Blasticidin S, Neomycin or Hygromycin-resistant genes. pMD2.G (Addgene #12259) and psPAX2 (Addgene #12260) were used for lentiviral packaging. pMD2.G and gag/pol (Addgene #14887) were used for retroviral packaging. All gRNA and gene information is provided in Supplementary Data 2.

LDTS of perilipin 1 was obtained from mouse Plin1 cDNA (Dharmacon, Clone Id:4166741) and LDTS of mouse perilipin 5, 4BNC and human PNPLA5 were synthesized (gBlocks, Integrated DNA technologies). eLIR and LIR mut fragments were obtained by PCR using primers containing mutations. PCR template was a plasmid containing human OPTN (Addgene #27052). PCR fragments were inserted into backbone plasmids using In-Fusion HD Cloning Kit (Takara) or NEBuilder HiFi DNA Assembly Master Mix (NEB). For AAV generation, mCherry, LDTS, and eLIR or LIR mut PCR fragments were inserted in the NotI-BamHI site of pAAV TBG plasmid (Adddgene #105535). The resulting plasmids were verified by sequencing.

### Palmitate solution

Stock solutions were prepared as follows: palmitic acid (Sigma-Aldrich) was dissolved in 75% ethanol at 70 °C at a final concentration of 300 mM. Aliquots of stock solutions were complexed with fatty-acid-free BSA (10% solution in 150 mM NaCl; Sigma-Aldrich) by stirring for 1 h at 37 °C. The final molar ratio of fatty acid:BSA was 5:1. The final ethanol concentration of stock solution was 1.5% (vol:vol). All control conditions included a solution of vehicle (ethanol:H_2_O) mixed with fatty-acid-free BSA in NaCl solution at the same concentration as the palmitate solution^76^.

### Virus production

To produce lentiviruses and retroviruses, 6-well plates of 70% confluent Lenti-X 293T(Clontech) cells were transfected with 1.5 μg of transfer vector, 0.5 μg pMD2.G and 1.0 μg of psPAX2 for lentivirus or 1.0 μg of gag/pol for retrovirus using Fugene HD (Promega) according to the manufacturer’s instructions. Supernatant was collected after 48 h and frozen at –80 °C. AAV packaging for LDTS-eLIR and LDTS-LIR mut were performed with AAV8 serotype at the University of Pennsylvania Vector Core (#V6014S; 8.462e13 GC/ml, #V6016S; 1.069e14 GC/ml, respectively). AAV to express Cre in the liver was purchased from Addgene (#107787-AAV8).

### Electron microscopy

HepG2 hepatocytes were trypsinized, washed, and fixed with 2.5% (vol/vol) glutaraldehyde, 2.0% (vol/vol) paraformaldehyde in 0.1 M sodium cacodylate buffer, pH 7.4, overnight at 4 °C. EM studies were performed on a JEM-1010 microscope at the University of Pennsylvania Electron Microscopy Resource Laboratory. Livers were fixed in 2% glutaraldehyde with 0.1 mM phosphate buffer (pH 7.2) for 24 h at 4 °C and postfixed in 2% osmium tetroxide with 0.1 mM phosphate buffer (pH 7.2) for 120 min at 4 °C and then serially dehydrated in ethanol and embedded in epoxy resin. Sections were cut on an LKB ultramicrotome and consecutive ultrathin sections were mounted on copper grids. Ultrathin sections were stained with 3% uranyl acetate and 0.2% lead citrate. Examinations were conducted with an electron microscope (JEM-2010 JOEL) in Hanaichi UltraStructure Reserch Institute.

### Oil red O staining

The Oil Red O staining was performed by fixing HepG2 cells in 4% paraformaldehyde and then staining with Oil Red O for 15 min. The samples were washed with 60% isopropanol for a few seconds, followed by three PBS washes. Analysis of stain-positive regions was performed using image J (National Institutes of Health, Bethesda, MD).

### Histopathological analysis

Mice were killed and liver, kidney, skeletal muscle, and WAT were fixed with 4% PFA overnight. Then fixed tissues were embedded in OCT or paraffin. The paraffin sections were used for hematoxylin and eosin (HE) staining and picrosirius red staining. The OCT sections were used for oil red O (Sigma-Aldrich, O0625) staining and immunohistochemistry (IHC) staining of αSMA. Analysis of stain-positive regions and adipocyte size was performed using ImageJ (National Institutes of Health, Bethesda, MD).

### RNA sequencing

Total RNA was isolated from the livers using TRIzol (Life Technologies) and Direct-zol RNA MiniPrep (Zymo Research Corporation) according to the manufacturer’s protocol. The library preparation was performed using a TruSeq-stranded mRNA sample prep kit (Illumina) according to the manufacturer’s instructions. Sequencing was performed on an Illumina NOVASeq 6000 platform in a 100 bp paired-end mode.

### Real-time PCR

RNA was isolated from livers using TRIzol (Life Technologies) and Direct-zol RNA MiniPrep (Zymo Research Corporation) according to the manufacturer’s instructions. Using the PrimeScript RT Master Mix (Takara Bio), we reverse transcribed total RNA. The cDNA was amplified by primers in a 10 µl reaction using KAPA SYBR FAST (Kapa Biosystems). We calculated mRNA using a ∆∆CT relative to the average of the housekeeping genes GAPDH expression. All primers and gene information were provided in Supplementary Data 3.

### Immunoblot

Total protein concentration of cell or liver lysate was determined using the Lowry assay (Bio-Rad Laboratories, 5000112JA). Equal amounts of protein were loaded onto Tris-glycine sodium dodecyl sulfate-polyacrylamide gels, separated by electrophoresis, and then the proteins were transferred onto polyvinylidene difluoride membranes (Millipore, IPVH00010). Membranes were then probed with anti-α-SMA (Abcam, ab5694, 1:100 for IHC, 1:1000 for WB), anti-ATG5 (Santa Cruz Biotechnology, sc133158, 1:500 for WB), anti-S6 Ribosomal Protein (CST, 2217, 1:1000 for WB), anti-Phospho-S6 Ribosomal Protein (Ser235/236) (CST, 2211, 1:1000 for WB), anti-ATGL (CST, 2138, 1:1000 for WB), and anti-β-actin (Sigma, A2228, 1:5000 for WB) antibodies followed by incubation with HRP-conjugated secondary antibodies (CST, 7076S/7074S, 3000:1 for WB). Immunolabeled bands were detected by chemiluminescence using the Clarity Western ECL substrate (Bio-Rad Laboratories,1705060) and the Clarity MAX Western ECL substrate (Bio-Rad Laboratories,1705062). Densitometric analysis of band intensity was performed using ImageJ (National Institutes of Health, Bethesda, MD).

### Biochemical assays of tissues

Lipids of tissue, serum exosome, cell lysate, and supernatant were extracted by the Folch method^77^. Triglyceride (TG) and non-esterified fatty acid (NEFA) concentrations were measured using test kits (LabAssay™ Triglyceride Kit, LabAssay™ NEFA Kit: Wako), according to the manufacturer’s instructions.

### Serum lipid profiling

Cholesterol and triglycerides in lipoproteins were analyzed by HPLC at Skylight Biotech (Akita, Japan)^78^.

### Fatty acid oxidation (FAO) activity assay

FAO enzyme activities were measured using FAO Assay Kit (Biomedical Research Service Center, E-141), according to the manufacturer’s instructions^79^. All samples were harvested using 1× Cell Lysis Solution. Protein concentration of the samples was assessed with a Lowry assay and normalized to 1 mg/ml. Add 20 µl of each sample to a plain 96-well plate placed on ice in duplicate. Then, swiftly add 50 µl control solution to one set of wells and 50 µl reaction solution to the other set of wells. Mix contents by gentle agitation for 10 s. Cover plate and keep in a non-CO_2_ incubator at 37 °C for 30 min. The plate was read at optical density of 492 nm (OD 492) with a microplate reader (BIO-RAD, iMark). Subtract control well reading from reaction well reading for each sample. FAO activity in IU/l unit was determined by multiplying OD by 12.96.

### Extracellular vesicles isolation

Exosome isolation from serum sample was performed using MagCapture Exosome Isolation Kit PS (Wako, 293-77601), according to the manufacturer’s instructions^80^. Magnetic beads coated with Tim4 protein (which binds to Phosphatidylserine on the membrane surface of extracellular vesicles) enable purification of highly purified EVs.

### BODIPY staining

For BODIPY 494/503 (4,4-Difluoro-1,3,5,7,8-Pentamethyl-4-Bora-3a,4a-Diaza-s-Indacene, Thermo Fisher Scientific, D3922) staining, cells were washed and stained with 2 µm BODIPY staining solution for 15 min at 37 °C. Cells were then washed, harvested by trypsinization, and resuspended with FACS buffer (PBS containing 2% FBS and 20 mM HEPES). Stained cells were analyzed on Attune NxT Flow Cytometer (Thermo Fisher Scientific) and analysis was done with FlowJo (Treestar)^81^.

### LysoSensor

For LysoSensor Green DND-189 (Thermo Fisher Scientific, L7535) staining, cells were washed and stained with 50 nM LysoSensor for 30 min at 37 °C. Cells were then washed, harvested by trypsinization, and resuspended with FACS buffer (PBS containing 2% FBS and 20 mM HEPES). Stained cells were analyzed on Attune NxT Flow Cytometer (Thermo Fisher Scientific) and analyzed with FlowJo (Treestar)^82^.

### Lysosomal acid lipase assay

This assay was performed using LysoLive Lysosomal Acid Lipase Assay Kit (Abcam, ab253380). Briefly, lipophagy-induced HepG2 cells were incubated LipaGreen for 8 h at 37 °C. Cells were then washed, harvested by trypsinization, and resuspended with FACS buffer (PBS containing 2% FBS and 20 mM HEPES). Stained cells were analyzed on Attune NxT Flow Cytometer (Thermo Fisher Scientific) and analyzed with FlowJo (Treestar).

### Lysosomal exocytosis analysis

To assess lysosomal exocytosis, we used LAMP1 expression on the membrane surface^83^. Cells were harvested by trypsinization, washed, and incubated with anti-LAMP1 antibodies (Biolegend, 328606, 1:50) for 20 min on ice. Cells were then washed and resuspended with FACS buffer. Stained cells were analyzed on Attune NxT Flow Cytometer (Thermo Fisher Scientific) and analysis was done with FlowJo (Treestar).

### Fura-2 Ca^2+^ imaging

293T cells expressing TRPML1 on the membrane surface were prepared, and were plated onto glass coverslips. Cells were loaded with 5 µM Fura-2 AM (DOJINDO, 343-05401) in the culture medium at 37 °C for 1 h. The extracellular bath solution (Tyrode’s solution) contained 153 mM NaCl, 5 mM KCl, 2 mM CaCl_2_, 1 mM MgCl_2_, 20 mM HEPES and 10 mM glucose (pH 7.4). The Low pH Tyrode solution contained 150 mM Na-gluconate, 5 mM KCl, 2 mM CaCl_2_, 1 mM MgCl_2_, 10 mM glucose, 10 mM HEPES, and 10 mM MES (pH 4.6). Oleic acid (Sigma-Aldrich) and fatty acid mixture (Sigma-Aldrich, L0288) were used as fatty acids. ML-SA1 (10 µM, TRPML agonist, Wako, 131-18531) were used as positive controls to induce Ca^2+^ release from lysosome and acidic stores. All bath solutions were applied via a perfusion system to achieve a complete solution exchange within a few seconds. Cells were recorded on the stage of an inverted microscope (IX-73, Olympus, Tokyo, Japan; 20 × 0.7 NA UCPlanFLN20 x PH) with continuous perfusion (RC-27L, Warner Instruments, Hamden, CT, USA). Cytosolic free Ca^2+^ concentrations were measured by dual-wavelength Fura-2 microfluorometry with excitation at 340/380 nm and emission at 510 nm. The ratio image was calculated and acquired using an sCMOS camera (ORCA-Flash4.0, Hamamatsu Photonics, Tokyo, Japan) and the HCImage software (Hamamatsu Photonics)^44^.

### Intraperitoneal glucose tolerance test (IPGTT) and intraperitoneal insulin tolerance test (IPITT)

IPGTT and ITT were performed on 16 weeks old mice two weeks after injection of AAV. After fasting, the baseline blood glucose level was measured by tail vein puncture. For IPGTT, mice were fasted for 15 h and a solution of 20% glucose (2 g/kg body weight) was administered intraperitoneally. After glucose administration, blood samples were collected from the tail vein at 15, 30, 60, and 120 min. For IPITT, mice were fasted for 5 h. Insulin-R(Eli-Lilly) was intraperitoneally injected (1 U/kg body weight) and blood samples from the tail vein were collected at 15, 30, 60, and 120 min after insulin injection. Glucose levels were evaluated with Glutentmint (Sanwa Kagaku Kenkyusho)^76^.

### Fasting plasma insulin and homeostatic model assessment of insulin resistance (HOMA-IR)

The blood samples collected after 15 h of fasting were used for the quantification of plasma insulin level with an enzyme-linked immunosorbent assay (ELISA), according to the manufacturer’s recommendations (MIoBS, M1104). HOMA-IR was estimated from fasting glucose and insulin as follows:

$${{{{{{\rm{HOMA}}}}}}}-{{{{{{\rm{IR}}}}}}}=\frac{{{{{{\rm{Fasting}}}}}}\; {{{{{\rm{insulin}}}}}}\,({{\upmu }}{{{{{\rm{IU}}}}}}/{{{{{\rm{mL}}}}}})\times {{{{{\rm{Fasting}}}}}}\; {{{{{\rm{glucose}}}}}}\,({{{{{\rm{mg}}}}}}/{{{{{\rm{dL}}}}}})}{405}$$

$${{{{{\rm{Insulin}}}}}}\,({{\upmu }}{{{{{\rm{IU}}}}}}/{{{{{\rm{mL}}}}}})={{{{{\rm{Insulin}}}}}}\,({{{{{\rm{ng}}}}}}/{{{{{\rm{ml}}}}}})\times 26$$

### Compound library screening

HepG2 cells expressing GFP-mCherry-LDTS and nuclear TagBFP were generated as lipophagy reporter cells. Clinically-approved or validated ~3500 compound library was provided as Plate on Demand (POD) by Drug Discovery Initiative in The University of Tokyo. Total of 5000 reporter cells were seeded by Multidrop 384 dispenser (ThermoFisher) in POD 384 well plate. After 24 h-culture, cells were analyzed with IN Cell Analyzer 2200. IN Cell Analyzer workstation was used to count TagBFP positive dots as cell number and measure total fluorescence of GFP and mCherry in mCherry-positive regions. Lipophagy score was calculated by (mCherry total fluorescence - GFP total fluorescence) in mCherry positive area and normalized with corresponding cell number. A DMSO-treated plate was used to correct position artifacts. For each plate, the raw value of lipophagy score was converted according to the value of DMSO-treated plat for all wells. Robust Z-scores were calculated using the following equation.

$${{{{{{\rm{Robust}}}}}}\; {{{{{\rm{Z}}}}}}\; {{{{{\rm{score}}}}}}}=\frac{{{{{{\rm{Converted}}}}}}\; {{{{{\rm{data}}}}}}{{{{{\rm{sample}}}}}}-{{{{{\rm{Median}}}}}}\; {{{{{\rm{of}}}}}}\; {{{{{\rm{converted}}}}}}\; {{{{{\rm{data}}}}}}{{{{{\rm{all}}}}}}\; {{{{{\rm{sample}}}}}}}{{{{{{\rm{Converted}}}}}}\; {{{{{\rm{robust}}}}}}\; {{{{{\rm{standard}}}}}}\; {{{{{\rm{deviation}}}}}}{{{{{\rm{all}}}}}}\; {{{{{\rm{sample}}}}}}}$$

### Statistical analyses

All data were expressed as mean ± SD. No statistical methods were used to predetermine sample size. Sample size was based on experimental feasibility and sample availability. Samples were processed in random order. Comparisons between the two groups were analyzed using the two-sided unpaired *t*-test. One-way ANOVA followed by Tukey’s or Dunnett’s multiple comparison test was used for multiple group comparisons using GraphPad Prism software version 9. *P* < 0.05 was considered statistically significant.

### Reporting summary

Further information on research design is available in the Nature Portfolio Reporting Summary linked to this article.

## Supplementary information

Supplementary information

Description of Additional Supplementary Files Document

Supplementary Data 1

Supplementary Data 2

Supplementary Data 3

Reporting Summary

## Data Availability

RNA-seq data have been deposited at GEO under accession code GSE185911. Source data are provided with this paper. Some plasmids are deposited in Addgene (#189000 - #189006). Other all unique/stable reagents generated in this study are available with a completed Materials Transfer Agreement.

## Source data

Source Data
