# Supplementary material for: Liver lipophagy ameliorates nonalcoholic steatohepatitis through extracellular lipid secretion
Source: Nat Commun. 2023 Jul 13;14:4084. doi: 10.1038/s41467-023-39404-6 (PMC10344867; doi:10.1038/s41467-023-39404-6)
Supplement: Supplementary file 2 — Description of Additional Supplementary Files Document [file 41467_2023_39404_MOESM2_ESM.pdf]

Description of Additional Supplementary Files

Supplementary Data 1: The list of compounds showing high score of robust Z for lipophagy induction.

Supplementary Data 2: The sequence information of gRNAs

Supplementary Data 3: The sequence information of PCR primers
