# Supplementary material for: Liver lipophagy ameliorates nonalcoholic steatohepatitis through extracellular lipid secretion
Source: Nat Commun. 2023 Jul 13;14:4084. doi: 10.1038/s41467-023-39404-6 (PMC10344867; doi:10.1038/s41467-023-39404-6)
Supplement: Supplementary file 6 — Reporting Summary [file 41467_2023_39404_MOESM6_ESM.pdf]

nature portfolio

Corresponding author(s): Atsushi Hoshino
Zoltan Arany

Last updated by author(s): May 15, 2023

Reporting Summary

Nature Portfolio wishes to improve the reproducibility of the work that we publish. This form provides structure for consistency and transparency in reporting. For further information on Nature Portfolio policies, see our Editorial Policies and the Editorial Policy Checklist.

Statistics

For all statistical analyses, confirm that the following items are present in the figure legend, table legend, main text, or Methods section.

n/a	Confirmed	
☐	☒	The exact sample size (*n*) for each experimental group/condition, given as a discrete number and unit of measurement
☐	☒	A statement on whether measurements were taken from distinct samples or whether the same sample was measured repeatedly
☐	☒	The statistical test(s) used AND whether they are one- or two-sided<br>*Only common tests should be described solely by name; describe more complex techniques in the Methods section.*
☒	☐	A description of all covariates tested
☒	☐	A description of any assumptions or corrections, such as tests of normality and adjustment for multiple comparisons
☐	☒	A full description of the statistical parameters including central tendency (e.g. means) or other basic estimates (e.g. regression coefficient) AND variation (e.g. standard deviation) or associated estimates of uncertainty (e.g. confidence intervals)
☐	☒	For null hypothesis testing, the test statistic (e.g. *F*, *t*, *r*) with confidence intervals, effect sizes, degrees of freedom and *P* value noted<br>*Give P values as exact values whenever suitable.*
☒	☐	For Bayesian analysis, information on the choice of priors and Markov chain Monte Carlo settings
☒	☐	For hierarchical and complex designs, identification of the appropriate level for tests and full reporting of outcomes
☒	☐	Estimates of effect sizes (e.g. Cohen's *d*, Pearson's *r*), indicating how they were calculated

Our web collection on statistics for biologists contains articles on many of the points above.

Software and code

Policy information about availability of computer code

Data collection	No software was used
Data analysis	GraphPad Prism 9 for statistical analysis<br>FlowJo v10 for flow cytometry analysis<br>image J 1.52p for microscopy images analysis

For manuscripts utilizing custom algorithms or software that are central to the research but not yet described in published literature, software must be made available to editors and reviewers. We strongly encourage code deposition in a community repository (e.g. GitHub). See the Nature Portfolio guidelines for submitting code & software for further information.

Data

Policy information about availability of data

All manuscripts must include a data availability statement. This statement should provide the following information, where applicable:

- Accession codes, unique identifiers, or web links for publicly available datasets
- A description of any restrictions on data availability
- For clinical datasets or third party data, please ensure that the statement adheres to our policy

RNA-seq data have been deposited at GEO under accession code GSE185911 (https://www.ncbi.nlm.nih.gov/geo/query/acc.cgi?acc=GSE185911).

Research involving human participants, their data, or biological material

Policy information about studies with human participants or human data. See also policy information about sex, gender (identity/presentation), and sexual orientation and race, ethnicity and racism.

Reporting on sex and gender	Randomization was stratified by sex to ensure a balanced design. Accordingly the analyses were adjusted for sex.
Reporting on race, ethnicity, or other socially relevant groupings	Race, ethnicity, or other socially relevant groups were not considered in this study design.
Population characteristics	The subjects were selected 1:1 against case control according to digoxin treatment, with age(71.3±9.6), sex(male:62.5%), BMI(21.3±3.1), and diagnosis for atrial fibrillation(75.0%) as matching criteria.
Recruitment	Opt-out method was used to obtain consent for this study for all patients who underwent abdominal ultrasound examinations between 2010/1/4 and 2020/12/28 at Kyoto Prefectural University of Medicine Hospital. A description of the research and contact information was made available on the website. It was explained that they were free to opt out of participation in the study by phone or through the website.
Ethics oversight	Clinical Research Review Committee in Kyoto Prefectural University of medicine (CRB5200001, ERB-C-2104)

Note that full information on the approval of the study protocol must also be provided in the manuscript.

Field-specific reporting

Please select the one below that is the best fit for your research. If you are not sure, read the appropriate sections before making your selection.

☒ Life sciences ☐ Behavioural & social sciences ☐ Ecological, evolutionary & environmental sciences

For a reference copy of the document with all sections, see nature.com/documents/nr-reporting-summary-flat.pdf

Life sciences study design

All studies must disclose on these points even when the disclosure is negative.

Sample size	No statistical methods were used to predetermine sample size. Sample size was based on experimental feasibility, sample availability, and N necessary to obtain definitive results.
Data exclusions	No data was excluded.
Replication	cell culture experiments were performed in biological 3-4 replication. Mouse experiments were done in n=6-9 per group. All attempts at replication were successful.
Randomization	Animals and samples were randomly sorted into experimental groups.
Blinding	Mouse experimenters were blinded. Other studies were not because knowledge of this information was essential to conduct the studies.

Reporting for specific materials, systems and methods

We require information from authors about some types of materials, experimental systems and methods used in many studies. Here, indicate whether each material, system or method listed is relevant to your study. If you are not sure if a list item applies to your research, read the appropriate section before selecting a response.

Materials & experimental systems

n/a	Involved in the study	
☐	☒	Antibodies
☐	☒	Eukaryotic cell lines
☒	☐	Palaeontology and archaeology
☐	☒	Animals and other organisms
☒	☐	Clinical data
☒	☐	Dual use research of concern
☒	☐	Plants

Methods

n/a	Involved in the study	
☒	☐	ChIP-seq
☐	☒	Flow cytometry
☒	☐	MRI-based neuroimaging

Antibodies

Antibodies used	mouse anti-αSMA (Abcam, ab5694, 1:100 for IHC, 1:1000 for WB)<br>mouse anti-ATG5 (Santa Cruz Biotechnology, sc133158, 1:500 for WB)<br>mouse anti-β actin (Sigma, A2228, 1:5000 for WB)<br>mouse anti-LAMP1 (Biolegend, 328606, 1:50 for FCM)<br>rabbit anti-S6 Ribosomal Protein (CST, 2217, 1:1000 for WB)<br>rabbit anti-Phospho-S6 Ribosomal Protein (Ser235/236) (CST, 2211, 1:1000 for WB)<br>rabbit anti-ATGL (CST, 2138, 1:1000 for WB)<br>anti-mouse-IgG antibody conjugated to HRP-linked antibody (CST, 7076S, 3000:1 for WB)<br>anti-rabbit-IgG antibody conjugated to HRP-linked antibody (CST, 7074S, 3000:1 for WB)
Validation	The antibodies that have been validated by the suppliers for specific purposes (for example, western blot) were purchased for our experiment. In addition, for key essential antibodies such as LAMP1 and ATG5 antibodies, we further validated the antibodies by FCM or western blotting upon depletion of the proteins.<br><br>mouse anti-αSMA (Abcam, ab5694)<br>Suitable for: WB, IHC(P)<br>https://www.abcam.co.jp/products/primary-antibodies/alpha-smooth-muscle-actin-antibody-ab5694.html<br><br>mouse anti-ATG5 (Santa Cruz Biotechnology, sc133158)<br>Suitable for: WB, IP, IF, IHC(P), ELISA<br>https://www.scbt.com/ja/p/atg5-antibody-c-1<br><br>mouse anti-β actin (Sigma, A2228)<br>Suitable for: WB, IHC, ICC, Ab Array<br>https://www.sigmaaldrich.com/JP/ja/product/sigma/a2228<br><br>mouse anti-LAMP1 (Biolegend, 328606)<br>Suitable for: FCM<br>https://www.biolegend.com/ja-jp/search-results/fitc-anti-human-cd107a-lamp-1-antibody-4966<br><br>rabbit anti-S6 Ribosomal Protein (CST, 2217)<br>Suitable for: WB, IF, IHC(P)<br>https://www.cellsignal.jp/products/primary-antibodies/s6-ribosomal-protein-5g10-rabbit-mab/2217<br><br>rrabbit anti-Phospho-S6 Ribosomal Protein (Ser235/236) (CST, 2211)<br>Suitable for: WB, IF, IHC(P), IP<br>https://www.cellsignal.jp/products/primary-antibodies/phospho-s6-ribosomal-protein-ser235-236-antibody/2211<br><br>rabbit anti-ATGL (CST, 2138)<br>Suitable for: WB, IF, IP<br>https://www.cellsignal.jp/products/primary-antibodies/atgl-antibody/2138

Eukaryotic cell lines

Policy information about cell lines and Sex and Gender in Research

Cell line source(s)	HepG2 (ATCC, HB-8065), 3T3L1 (ATCC, CL-173), Lenti-X 293T (Clontech, 632180)
Authentication	Cell lines were authenticated with morphology, karyotyping, and PCR based approaches by ATCC or Clontch.
Mycoplasma contamination	The cell lines were tested for potential mycoplasma contamination and confirmed that they are mycoplasma negative.
Commonly misidentified lines (See ICLAC register)	No commonly misidentified cell lines were used.

Animals and other research organisms

Policy information about studies involving animals; ARRIVE guidelines recommended for reporting animal research, and Sex and Gender in Research

Laboratory animals	This study used 8 weeks old male wild-type and ATG5flox/flox mice (C57BL/6J) for NASH model.
Wild animals	The study did not involve wild animals.
Reporting on sex	Only males were used in this study. Sex was not considered in this study design as we did not observe sex differences in the field of lipophagy in the past.
Field-collected samples	The study did not involve samples collected from the field.

Ethics oversight	All mouse experiments were approved by the Animal Care and Use Committee of the Kyoto Prefectural University of Medicine (M2020-59).

Note that full information on the approval of the study protocol must also be provided in the manuscript.

Flow Cytometry

Plots

Confirm that:

- [x] The axis labels state the marker and fluorochrome used (e.g. CD4-FITC).
- [x] The axis scales are clearly visible. Include numbers along axes only for bottom left plot of group (a 'group' is an analysis of identical markers).
- [x] All plots are contour plots with outliers or pseudocolor plots.
- [x] A numerical value for number of cells or percentage (with statistics) is provided.

Methodology

Sample preparation	HepG2 cells were suspended and incubated with Alexa488-conjugted anti Lamp1 antibody. LysoSensor Green DND-189 and LipaGreen was incubated for 20 min and 8 hr before cell sorting, respectively.
Instrument	Attune NxT Flow Cytometer (Invitrogen)
Software	FlowJo v10 (FlowJo, LLC)
Cell population abundance	Not confirmed.
Gating strategy	Gating was performed according to Lamp1-Alexa488 signal strength.

- [ ] Tick this box to confirm that a figure exemplifying the gating strategy is provided in the Supplementary Information.
